# Open questions in ethics of virtual training

**DOI:** 10.1192/j.eurpsy.2025.218

**Published:** 2025-08-26

**Authors:** M. Lamb

**Affiliations:** Informatics, University of Skövde, Skövde, Sweden

## Abstract

**Abstract:**

In recent years, developments in Generative artificial intelligence (genAI) have opened new possibilities for continuous education that were recently only science fiction. However, genAI introduces several ethical considerations and ethical issues continue to appear. These include privacy, ownership, accuracy, bias, psychological impact, and environmental impact. In this workshop we will consider where these ethical issues might specifically intersect with the development and implementation of genAI in training professional conversational skills in the context of professional mental health support. Understanding these ethical issues is important for ensuring positive and sustainable impact on society and individual health. As a part of this workshop, we consider theoretical issues, but we will also look at methods to ensure the development of ethical and trustworthy genAI systems, including transparency and human-in-the-loop methods, along with quality and risk management systems. Moreover, when considering worker health, it is will be important that future work empowers workers to continue to develop in their field, without introducing additional harms such as accessibility problems or exposure to harmful imagery or interactions.

**Disclosure of Interest:**

None Declared

